# Kälteagglutininerkrankung

**DOI:** 10.1007/s00108-025-01926-0

**Published:** 2025-07-21

**Authors:** Alexander Röth, Kersten Borchert, Carla Dorn, Moritz Kleemiß, Sixten Körper, Stephanie Mayer, Philippe Schafhausen, Karin G. Schrenk, Peter Bramlage, Frauke Theis

**Affiliations:** 1 Klinik für Hämatologie und Stammzelltransplantation, Klassische Hämatologie und Hämostaseologie, Westdeutsches Tumorzentrum, Universitätsklinikum Essen, Universität Duisburg-Essen, Hufelandstraße 55, 45147 Essen, Deutschland; 2 Klinik für Hämatologie und Onkologie, AMEOS Klinika Aschersleben und Staßfurt, Akademisches Lehrkrankenhaus der Universität Magdeburg, Aschersleben, Deutschland; 3 Klinikum Fürth, Universitätsklinikum Bayreuth, Fürth, Deutschland; 4 Universitätsklinikum Bonn, Bonn, Deutschland; 5 DRK-Blutspendedienst Baden-Württemberg – Hessen, Mannheim, Deutschland; 6 Klinik und Poliklinik für Innere Medizin III, Universitätsklinikum Regensburg, Regensburg, Deutschland; 7 Klinik und Poliklinik für Onkologie, Hämatologie und Knochenmarktransplantation mit der Abteilung für Pneumologie, Universitäres Cancer Center Hamburg (UCCH), Universitätsklinikum Hamburg-Eppendorf, Hamburg, Deutschland; 8 Abteilung Hämatologie und Internistische Onkologie, Klinik für Innere Medizin II, Universitätsklinikum Jena, Jena, Deutschland; 9 IPPMed – Institut für Pharmakologie und Präventive Medizin, Cloppenburg, Deutschland; 10 Medizinische Klinik III für Hämatologie, Onkologie und Palliativmedizin, Zentrum für Innere Medizin, Universitätsmedizin Rostock, Rostock, Deutschland; 11 Institut für Klinische Transfusionsmedizin und Immungenetik, Universität Ulm, Ulm, Deutschland

**Keywords:** Kälteagglutininerkrankung/Prognose, B‑Lymphozyten, Proteine des Komplementsystems, Sutimlimab, Coombs-Test, Cold agglutinin disease/prognosis, B‑lymphocytes, Complement system proteins, Sutimlimab, Coombs test

## Abstract

**Hintergrund:**

Die Kälteagglutininerkrankung („cold agglutinin disease“ [CAD]) ist eine seltene, aber klinisch eindrucksvolle Erkrankung mit hohem Leidensdruck und einem Risiko für schwerwiegende thromboembolische Komplikationen.

**Ziel:**

Der vorliegende Übersichtsbeitrag soll eine prägnante, klinisch orientierte Zusammenfassung des aktuellen Kenntnisstands zur Erkrankung und zu den Therapieoptionen bieten.

**Ergebnisse:**

Die Diagnose erfordert den Nachweis einer chronischen Hämolyse, die Detektion von C3d im monospezifischen direkten Coombs- bzw. Antiglobulintest (DAT), den Nachweis von Kälteagglutininen mit einem Titer ≥ 1:64 bei 4 °C und den Ausschluss einer malignen Erkrankung oder relevanten Infektion. Therapeutische Optionen waren bislang die Vermeidung von Kälte, eine adäquate Hydratation bei hämolytischen Krisen, eine Thromboseprophylaxe sowie eine immunsuppressive Therapie mit Rituximab und/oder Zytostatika. Die einzige zugelassene Therapie ist eine Komplementinhibition mit Sutimlimab. Die Hämolyse spricht auf eine Inhibition des klassischen Komplementwegs durch den Anti-C1s-Antikörper Sutimlimab innerhalb von wenigen Tagen mit einem Abfall der Hämolyseparameter und einer Verbesserung der Fatigue an. Die Therapie erfordert einen vollumfänglichen Impfschutz gegen bekapselte Bakterien und gegebenenfalls eine überbrückende antibiotische Prophylaxe, bis dieser erreicht ist. Eine supportive Therapie, beispielsweise die Gabe von Folsäure, Vitamin B12 und Eisen bei Mangel, oder auch eine Kombination mit weiteren Therapiestrategien sollte gegebenenfalls erwogen werden. Daten zum Einsatz von Sutimlimab beim sekundären Kälteagglutininsyndrom (CAS) liegen bislang nicht vor.

**Schlussfolgerung:**

Die Kenntnis der spezifischen Klinik und der Laborveränderungen bei einer Kälteagglutininerkrankung mit der frühzeitigen Einleitung einer spezifischen und zielgerichteten Therapie oder aber auch mit der Überweisung an ein spezialisiertes Zentrum hat die Prognose der Erkrankung in den letzten Jahren deutlich verbessert und den Leidensdruck der Patienten reduziert.

## Abstract (en)

**Background:**

Cold agglutinin disease (CAD) is a rare but clinically impressive disease with a high level of disease burden and a risk of severe thromboembolic complications.

**Objective:**

This review article provides a concise, clinically oriented summary of the current knowledge on the disease and the treatment options.

**Results:**

The diagnosis requires the detection of a chronic hemolysis with demonstration of C3d in a monospecific direct Coombs or antiglobulin test (DAT), the detection of cold agglutinins with a titer ≥ 1:64 at 4 °C and the exclusion of a malignant disease or relevant infection. Treatment options are so far the avoidance of low temperatures, adequate hydration in hemolytic crises, thrombosis prophylaxis as well as immunosuppressive treatment with rituximab and/or cytostatic agents. The only approved treatment is complement inhibition with sutimlimab. The hemolysis responds to inhibition of the classical complement pathway with the anti-C1s antibody sutimlimab within a few days with a decrease of hemolysis parameters and an improvement of fatigue. The treatment requires a comprehensive vaccination against capsulated bacteria and if necessary, a bridging antibiotic prophylaxis until this has been achieved. Supplementary treatment, such as administration of folic acid, vitamin B12 and iron in cases of deficiency or also a combination with other treatment strategies should be considered when necessary. Data on the use of sutimlimab in secondary cold agglutinin syndrome (CAS) are not yet available.

**Conclusion:**

Knowledge of the specific clinical and laboratory hallmark changes of CAD with the early initiation of specific and targeted therapy or also referral to specialized centers has significantly improved the prognosis of the disease in recent years and reduced the suffering of patients.

Hämolyse, Anämie, Abgeschlagenheit, Dyspnoe und Hämoglobinurie, unter Umständen auch in Kombination mit Akrozyanose, Raynaud-Phänomen, Livedo reticularis und Gangrän, weisen auf eine Autoimmunerkrankung hin, die als Kälteagglutininerkrankung („cold agglutinin disease“ [CAD]) erst in den letzten Jahren verstärkte Aufmerksamkeit erfahren hat. Sie ist komplizierend mit Thromboembolien und einer möglicherweise erhöhten Mortalität assoziiert. Neben Allgemeinmaßnahmen wie Schutz vor kalten Temperaturen zur Vermeidung hämolytischer Krisen basieren aktuelle Therapien auf einer Depletion der B‑Lymphozyten sowie einer Inhibition des klassischen Aktivierungswegs des Komplementsystems. Die Inhibition des klassischen Aktivierungswegs des Komplementsystems zur Behandlung dieser autoimmunhämolytischen Anämie (AIHA) hat sich als zuverlässig und wirksam erwiesen. Die Datenlage und der praktische klinische Einsatz sind Gegenstand dieser Übersicht.

## Definition

Die CAD ist eine eigenständige lymphoproliferative B‑Zell-Erkrankung (Internationale statistische Klassifikation der Krankheiten und verwandter Gesundheitsprobleme, 10. Revision, German Modification [ICD-10-GM 2024] D59.10; [1–4]). Sie ist abzugrenzen vom Kälteagglutininsyndrom („cold agglutinin syndrome“ [CAS]), das sekundär bei malignen Erkrankungen (vor allem B‑Zell-Lymphomen) oder akuten Infektionen (unter anderem mit *Mycoplasma pneumoniae*, Epstein-Barr-Virus oder „severe acute respiratory syndrome coronavirus type 2“ [SARS-CoV-2]) auftritt [1, 2]. Die Kälteagglutinine sind zu 90 % vom Typ Immunglobulin M κ (IgM κ; selten auch Immunglobulin G [IgG]) und binden an ein Oberflächenantigen von Erythrozyten (Antigen I) bei ≤ 37 °C [2, 4, 5]. Sie führen so zur Agglutination. Dieser IgM-Antigenkomplex aktiviert typischerweise den klassischen Aktivierungsweg des Komplementsystems mit konsekutiver komplementvermittelter Hämolyse [2, 6].

## Pathophysiologie

**Abb. 1:**
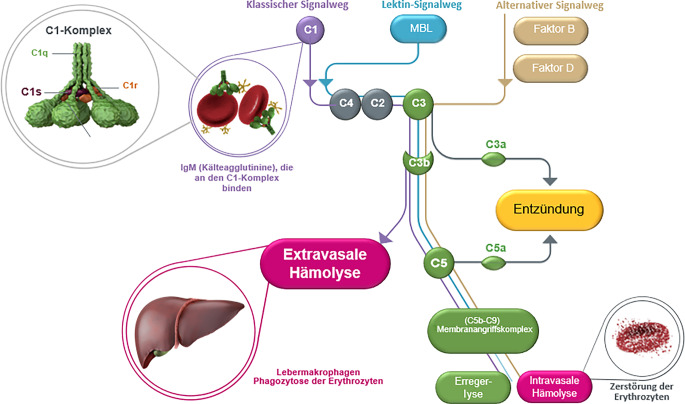
Pathophysiologie der Kälteagglutininerkrankung. *C1, C2, C3, …* Komplementfaktor 1, 2, 3, …, *IgM* Immunglobulin M, *MBL* mannosebindendes Lektin. (Nach Röth [6])

Kälteagglutinine werden von B‑Lymphozyten im Knochenmark und zum Teil von ausgereiften Plasmazellen gebildet. Es handelt sich dabei überwiegend um IgM-Antikörper (91 %), gefolgt von IgG (4,5 %) und Mischformen aus IgM und IgG (2,8 %; [7]). Durch die Bindung der Kälteagglutinine an Erythrozyten aktivieren sie den klassischen Aktivierungsweg des Komplementsystems mit C1q über C1r und C1s (Abb. 1). Diese Aktivierung resultiert zum einen in der Bildung der C3-Konvertase, mit Bindung von C‑Fragmenten (C3b) auf den Erythrozyten und konsekutiver chronischer extravasaler Hämolyse der komplementbeladenen Erythrozyten, vornehmlich in der Leber und zu einem geringen Anteil in der Milz. Zum anderen kann C3b an die C3-Konvertase binden und so die C5-Konvertase bilden. Dies führt zu einer Spaltung von C5. Das Spaltprodukt C5b bildet mit C6–C9 den Membranangriffskomplex (MAC), der dann zu einer unterschiedlich stark ausgeprägten intravasalen Hämolyse führen kann [6].

Lymphoproliferative Erkrankung und komplementabhängige Hämolyse sind mögliche Therapieansatzpunkte

Aufgrund der dargestellten Pathophysiologie stellen zum einen die lymphoproliferative Erkrankung (Ursache) und zum anderen auch die komplementabhängige Hämolyse (Komplikation) mögliche Ansatzpunkte für eine therapeutische Intervention dar [3].

## Diagnosestellung

Für die Diagnosestellung sind

- die Sicherung einer chronischen Hämolyse (Tab. 1),
- ein deutlicher Nachweis von C3d im monospezifischen direkten Coombs- bzw. Antiglobulintest (DAT),
- der Nachweis von Kälteagglutininen mit einem Titer ≥ 1:64 bei 4 °C und
- der Ausschluss einer malignen Erkrankung oder relevanten Infektion

wegweisend [8]. Ergänzend kann noch der Titer bei 4 °C, 22 °C, 30 °C und 37 °C bestimmt werden zur Festlegung der sogenannten Thermalamplitude [9]. Die Diagnose lässt sich bestätigen [4] durch den Nachweis von monoklonalem IgM κ im Serum (selten IgG, Immunglobulin A [IgA] oder λ), ein κ/λ-Verhältnis von > 3,5 (selten < 0,9) der monoklonalen B‑Lymphozyten-Population (im Knochenmark) mittels Immunphänotypisierung und gegebenenfalls ergänzend den Nachweis einer lymphoproliferativen B‑Zell-Erkrankung in der Knochenmarkhistologie, die Abwesenheit einer MYD88-L265P-Mutation und erniedrigte C4-Spiegel. Die IgM-Autoantikörper binden meist an das Antigen I auf den Erythrozyten. Wichtig ist die konsequente Lagerung bzw. der Transport der Proben bis zur Analyse bei Temperaturen zwischen 37 und 38 °C.

**Tab. 1:** Diagnostische Kriterien für eine Kälteagglutininerkrankung

	Kriterien	Vorgehen und Kommentar
Hauptkriterien	Chronische Hämolyse	Nachweis durch erhöhten Bilirubinwert, niedriges/nicht messbares Haptoglobin, hohen LDH-Wert und oft eine erhöhte Retikulozytenzahl/Retikulozytose
	Monospezifischer DAT stark positiv für C3d	–
	Kälteagglutinintiter ≥ 1:64 bei 4 °C	–
	Kein Anhalt für eine maligne Erkrankung/ein Lymphom oder eine relevante Infektion	–
Nebenkriterien	Monoklonale IgM κ im Serum (selten IgG, IgA oder λ)	Proben müssen bei 37–38 °C aufbewahrt werden, bis Plasma/Serum von Zellen/Clot getrennt ist
	κ/λ-Verhältnis > 3,5 (selten < 0,9) der B‑Lymphozytenpopulation im Knochenmark	Durchflusszytometrie im Knochenmark
	Nachweis einer lymphoproliferativen B‑Zell-Erkrankung in der histologischen Untersuchung	Knochenmarkbiopsie
	Abwesenheit einer MYD88-L265P-Mutation	Knochenmark
	C4-Spiegel	Typischerweise erniedrigt

*DAT* direkter Antiglobulintest, *IgA* Immunglobulin A, *IgG* Immunglobulin G, *IgM* Immunglobulin M, *LDH* Laktat-Dehydrogenase

## Klinische Präsentation

In der klinischen Präsentation unterscheidet man zwischen *komplementvermittelten* [4, 10–12] und *kälteagglutininvermittelten* [13–15] *Symptomen*. Typische komplementvermittelte Symptome umfassen

- Hämolyse mit hämolytischer Anämie,
- Abgeschlagenheit (Fatigue),
- Dyspnoe,
- Hämoglobinurie und
- Ikterus.

Kälteagglutininvermittelt sind zumeist temperaturabhängig reversible Symptome wie

- Akrozyanose (Blaufärbung der Akren),
- Raynaud-Phänomen (Erblassen von Fingern oder Zehen aufgrund von Vasospasmen),
- Livedo reticularis (Kältemarmorierung) und
- Gangrän.

## Differenzialdiagnosen

Die Differenzialdiagnose der CAD ist das CAS. Dieses tritt gelegentlich akut bei Kindern und Jugendlichen wie auch bei Erwachsenen im Kontext eines Virusinfekts auf, kann aber auch chronisch verlaufen, etwa bei Lymphomen [10, 16]. Kennzeichnend sind der plötzliche Beginn und die spontane Remission.

## Thrombosen und Mortalität

Die CAD ist mit einem erhöhten Risiko für Thromboembolien und möglicherweise auch mit einer erhöhten Mortalität assoziiert. Eine Beobachtungsstudie und Auswertungen von Daten aus Patientenregistern zeigen ein leicht erhöhtes Risiko für venöse Thromboembolien im Vergleich zur Normalbevölkerung oder zu gematchten Kontrollgruppen (Größenordnung 1,7- bis 3,1fach [7, 17, 18]). Arterielle Ereignisse sind dagegen in den meisten Datensätzen weniger stark mit der CAD assoziiert. Dabei wurde eine Korrelation des Ausmaßes der Anämie mit dem Thromboserisiko diskutiert, aber nicht in allen Datensätzen bestätigt [7, 17].

Die CAD ist mit einem erhöhten Risiko für Thromboembolien assoziiert

Daten zur Mortalität bei Patienten mit CAD zeigen ein unverändertes bis leicht erhöhtes Sterblichkeitsrisiko im Vergleich zur Normalbevölkerung insbesondere in den ersten 5 Jahren nach Erstdiagnose [7, 18, 19].

## Therapie

### Allgemeine Maßnahmen

Patienten mit CAD sollten sich konsequent vor kalten Temperaturen schützen, um keine zusätzlichen hämolytischen Krisen zu provozieren. Das wird auf der einen Seite durch warme Bekleidung erreicht (unter anderem warme oder aktiv wärmende Socken, Handschuhe und gegebenenfalls Gesichtsschutz), auf der anderen Seite durch die Vermeidung von kalten Getränken, Speiseeis, kalter Luft oder kalten Infusionen bzw. Transfusionen. Darüber hinaus sinnvoll ist die Supplementierung mit Folsäure (5 mg/Tag), gegebenenfalls auch mit Vitamin B12 und Eisen bei Mangelsituationen. Bakterielle Infektionen sollten frühzeitig und konsequent antibiotisch behandelt werden, um infektgetriggerte hämolytische Krisen zu vermeiden.

Bei der Behandlung einer CAD kann insbesondere in Akutsituationen die Transfusion von Erythrozytenkonzentraten notwendig sein. Während einer Transfusion oder auch Infusion sollten die Extremitäten möglichst warm gehalten werden. Es sollte ein spezieller Blut- bzw. Infusionswärmer verwendet werden. Ohne diese Maßnahmen besteht die Möglichkeit, dass die transfundierten Erythrozyten, die in der Regel bei 4 °C gelagert werden, in der Zirkulation agglutinieren. Zusätzlich wird die Erwärmung des Bedside-Tests zur Vermeidung falsch-positiver Ergebnisse empfohlen. Zudem sollte auch bei einer kritischen Hämolyse auf eine ausreichende Hydratation geachtet werden. Ebenso empfiehlt sich eine konsequente Thromboseprophylaxe in therapeutischen Dosierungen. Der Einsatz von Steroiden, alkylierenden Substanzen oder Interferon und eine Splenektomie sind nicht sinnvoll und haben sich als nicht oder kaum wirksam erwiesen [1, 2, 4, 5, 20, 21].

### Spezifische Therapien

Die Therapiestrategie bei CAD umfasst eine gegen B‑Zellen gerichtete Immunchemotherapie und neu die Inhibition des Komplementsystems mit dem monoklonalen Anti-C1s-Antikörper Sutimlimab.

Insbesondere in Akutsituationen kann die Transfusion von Erythrozytenkonzentraten notwendig sein

Zwischen 2004 und 2020 wurde eine Reihe von Studien veröffentlicht, die die Wirksamkeit von gegen B‑Zellen gerichteten Immunchemotherapien untersuchten. Untersucht wurde dabei die Wirksamkeit des gegen CD20 auf der Oberfläche von Lymphozyten gerichteten monoklonalen Antikörpers Rituximab [22, 23], des Proteasominhibitors Bortezomib [24] und der Kombinationen von Fludarabin (Purinanalogon, Hemmer der DNA-Synthese) oder Bendamustin (DNA-Alkylans) mit Rituximab [7, 25, 26]. Die höchsten Raten einer vollständigen Remission wurden mit der Kombination aus Bendamustin und Rituximab (40–53 %) erzielt, wobei die Remissionsdauer bis zu 66 bzw. 88 Monate betrug (Median noch nicht erreicht; [7, 25]). Die Toxizität der Therapien bei den in der Regel älteren Patienten ist mit Ausnahme der Kombination von Fludarabin mit Rituximab [26] tolerabel. Die Wirkstoffe sind aktuell jedoch nicht für die Therapie der CAD zugelassen.

Die Komplementinhibition kommt als therapeutische Strategie schon bei einer Reihe unterschiedlicher Erkrankungen zum Einsatz. Pegcetacoplan ist ein C3-Inhibitor, Eculizumab, Ravulizumab und Crovalimab sind C5-Inhibitoren, Iptacopan hemmt den Faktor B und Danicopan den Faktor D des alternativen Pfads zur Komplementaktivierung. Als C1-Inhibitor ist Sutimlimab klinisch am weitesten entwickelt und bereits für die Therapie zugelassen; alternative C1-Inhibitoren sind ANX005 und C1-INH [6].

### Patientenschulung

Es ist wichtig sicherzustellen, dass die Patienten die Wichtigkeit eines vollständigen Impfschutzes verstehen. Entsprechend sollten sie auf Symptome und Anzeichen von Infektionen achten und den behandelnden Arzt informieren. Im Alltag ist die strikte Meidung einer Kälteexposition wichtig. Hämolytische Krisen mit Hämoglobinurie werden oft als Hämaturie, beispielsweise bei Harnwegsinfektionen, fehlinterpretiert.

### Sutimlimab

Sutimlimab ist ein humanisierter monoklonaler Antikörper, der sich spezifisch gegen eine Untereinheit des Komplementfaktors 1 (C1s) des klassischen Aktivierungswegs richtet und mit den anderen Aktivierungswegen des Komplementsystems nicht interferiert. Er verhindert die komplementvermittelte extravasale (über C3b) und auch intravasale Hämolyse (über C3a und C5a sowie den MAC C5b–C9).

Die Wirksamkeit und Sicherheit von Sutimlimab wurde in zwei multizentrischen Phase-III-Studien untersucht:

- In der einarmigen CARDINAL-Studie [6] wurden 24 Patienten mit CAD und einer kürzlich (< 6 Monate zuvor) durchgeführten Transfusion über 26 Wochen behandelt und 2 Jahre nachverfolgt. Der kombinierte primäre Endpunkt aus Anstieg des Hämoglobin(Hb)-Werts, Transfusionsfreiheit und fehlender Notwendigkeit einer weiteren Therapie der CAD wurde in der CARDINAL-Studie von 54 % der Patienten erreicht (95%-Konfidenzintervall [KI] 32,8–74,4 %).
- Die randomisierte CADENZA-Studie [27] war placebokontrolliert und wurde bei Patienten mit CAD durchgeführt, die unabhängig von Transfusionen waren. Das Design war darüber hinaus weitgehend mit dem der CARDINAL-Studie vergleichbar. Der kombinierte primäre Endpunkt wurde unter einer Sutimlimabtherapie von 72,7 % der Patienten erreicht (95 %-KI 49,8–89,3 %; *p* < 0,001) im Vergleich zu 15,0 % (95 %-KI 3,2–37,9 %) in der Kontrollgruppe.

Klinisch relevante Veränderungen des Hb-Werts, des Bilirubinwerts und des Functional-Assessment-of-Chronic-Illness-Therapy(FACIT)-Fatigue-Scores wurden dabei bereits nach nur kurzer Therapiedauer erreicht. In der CARDINAL-Studie [6] kam es schon innerhalb der ersten Woche zu einem relevanten Anstieg des Hb-Werts um mehr als 1 g/dl auf ein stabiles und nachhaltiges Niveau von mehr als 11 g/dl. Der Bilirubinwert fiel bzw. normalisierte sich nach 1–2 Dosen Sutimlimab (innerhalb von 1 bis 3 Wochen) und der FACIT-Fatigue-Score stieg innerhalb der ersten Woche klinisch relevant um über 5 Punkte auf ein neues Plateau zwischen 40 und 45 an. Vergleichbare Veränderungen wurden auch in der Sutimlimabgruppe der CADENZA-Studie [27] beobachtet, während die Werte der Patienten im Kontrollarm unverändert blieben. Sutimlimab wurde dabei gut toleriert und die Morbidität entsprach weitgehend dem Profil einer älteren und komorbiden Patientenpopulation. Schwerwiegende Infektionen wurden nicht beobachtet.

### Therapiealgorithmus

Ein Konsensus zum Vorgehen bei symptomatischer CAD wurde zuletzt im Jahr 2020 von Jäger et al. [28] und auch von Röth [29] veröffentlicht. Er unterscheidet grundsätzlich zwischen asymptomatischen („watch and wait“) und symptomatischen CAD-Patienten. Kennzeichnend für letztere sind eine Anämie, der Bedarf für die Transfusion von Erythrozytenkonzentraten, Fatigue und Mikrozirkulationsstörungen. Therapeutisch stand seinerzeit Rituximab – möglicherweise in Kombination mit Bendamustin – im Vordergrund der Überlegungen, während in der Notfallsituation noch ein Plasmaaustausch (gegen Albumin als Ersatzflüssigkeit) oder eine Therapie mit Eculizumab empfohlen wurden [30]. Diese Empfehlungen gründeten auf begrenzter Evidenz; entsprechende Zulassungen bestanden und bestehen nicht. Durch die Daten der Studien CARDINAL [6] und CADENZA [27] und die konsekutive Zulassung von Sutimlimab ergeben sich in Zukunft Veränderungen der Empfehlung.

#### Essener Therapieschema für Sutimlimab

Im Rahmen einer Neueinstellung mit Sutimlimab sollten Schutzimpfungen gegen Meningokokken (MCV-ACWY und MenB), Pneumokokken (Pneumokokkenkonjugatimpfstoff PCV20) und *Haemophilus influenzae* Typ B (Act-Hib) erfolgen. Regelhaft wird hier bei simultanem Therapiebeginn und Impfung eine antibiotische Prophylaxe mit 2‑mal 1 Mio. Einheiten Penicillin V (Phenoxymethylpenicillin) pro Tag für die ersten 2 Wochen bis zum Erreichen eines wirksamen Impfschutzes empfohlen. Zusätzlich sind eine jährliche Grippeschutzimpfung sowie eine Impfung gegen SARS-CoV‑2 sinnvoll.

**Abb. 2:**
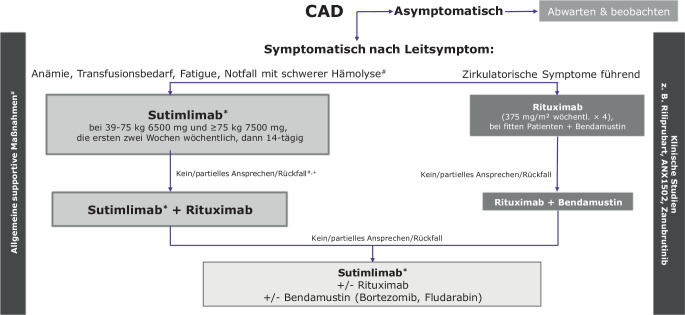
Therapiealgorithmus für die CAD. *Raute* Gegebenenfalls Plasmaaustausch diskutieren. *Paragrafenzeichen* Allgemeine supportive Maßnahmen, beispielsweise Vermeidung kalter Temperaturen, frühzeitige antibiotische Therapie bakterieller Infektionen, Supplementierung mit Folsäure, gegebenenfalls Vitamin B12 und Eisen (sofern ein Mangel vorliegt). *Asterisk* Zugelassen zur Behandlung der CAD. *Plus* Kritische Hinterfragung der Diagnose. *CAD* „cold agglutinin disease“ (Kälteagglutininerkrankung). (Modifiziert nach [28, 29])

Sutimlimab wird in Abhängigkeit vom Körpergewicht dosiert (Abb. 2). Dabei erhalten Patienten bei einem Körpergewicht zwischen 39 und 75 kg 6500 mg und ab einem Körpergewicht von 75 kg 7500 mg. Infusionen erfolgen bei Therapiebeginn im Abstand von einer Woche. Die Erhaltungstherapie erfolgt dann alle 2 Wochen mit einer Toleranz von ± 2 Tagen. Die Patienten werden nach Abschluss der Infusion und Nachspülung während der Aufsättigungsphase für 2 h, später während der Erhaltungstherapie für 1 h nachbeobachtet. Sollte sich die Erhaltungstherapie verzögern (> 17 Tage), muss eine erneute Aufsättigung erfolgen. Sutimlimab sollte insbesondere auch im Notfall bei nicht vorbehandelten Patienten mit schweren hämolytischen Krisen eingesetzt werden.

### Verlaufskontrolle

**Abb. 3:**
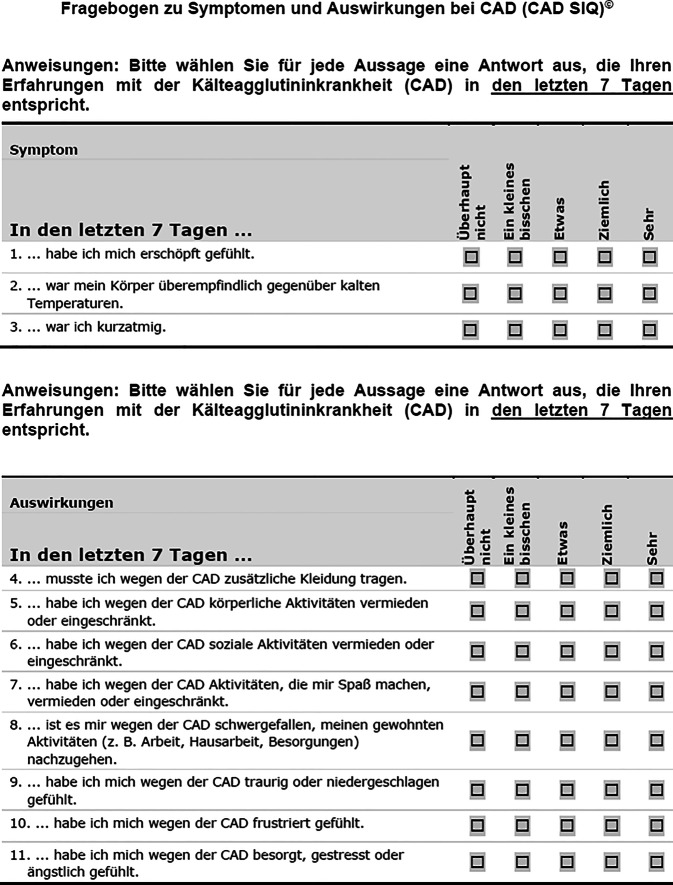
Symptome und Auswirkungen bei Kälteagglutininerkrankung (CAD-SIQ). *CAD* „cold agglutinin disease“ (Kälteagglutininerkrankung), *CAD-SIQ* Cold Agglutinin Disease Symptoms and Impact Questionnaire

Sinnvolle Kontrollen des Erkrankungsverlaufs und des Ansprechens auf die eingeleitete Therapie umfassen neben den Parametern der hämolytischen Aktivität wie Hb- und Bilirubinwerten und Retikulozyten auch Parameter der Komplementaktivierung bzw. des Komplementverbrauchs wie C3, C4, CH50 (Globaltest für die Gesamtkomplementaktivtät) und den monospezifischen DAT (Tab. 2). Weiterhin sollte auch die lymphoproliferative Erkrankung durch Bestimmung des quantitativen IgM überwacht werden. Mangelzustände können sich auch unter einer Komplementinhibition manifestieren. Regelmäßige Kontrollen des Ferritin‑, Folsäure- und Vitamin-B12-Spiegels sind daher sinnvoll. Eine Kontrolle der Fatigue mithilfe von Fragebögen (FACIT-Fatigue, CAD Symptoms and Impact Questionnaire [CAD-SIQ]; Abb. 3 im Anhang) wird insbesondere longitudinal empfohlen.

**Tab. 2:** Verlaufskontrollen der Kälteagglutininerkrankung unter Sutimlimab

Akut sinnvoll	Im Intervall sinnvoll
Blutbild und Hämolyseparameter (Bilirubin, LDH)	Folsäure/Vitamin B12/Ferritin Quantitatives IgM im Serum (sofern vorhanden) Monospezifischer DAT, Kälteagglutinintiter C4-Spiegel D‑Dimere ANA/ANCA Blutdruck

Probenmaterial muss bis zur Analyse bei 37–38 °C gehalten werden!

*ANA* antinukleäre Antikörper, *ANCA* antineutrophile zytoplasmatische Antikörper, *C4* Komplementfaktor 4, *DAT* direkter Antiglobulintest, *IgM* Immunglobulin M, *LDH* Laktat-Dehydrogenase

### Einschränkungen

Sutimlimab wird als Dauertherapie angewendet, mit Infusionen im Abstand von jeweils 2 Wochen, die regelhaft in einer medizinischen Einrichtung erfolgen sollten. Die häufigsten Nebenwirkungen sind

- Kopfschmerzen,
- Hypertonie,
- Harnwegsinfektionen,
- Infektion der oberen Atemwege,
- Nasopharyngitis,
- Übelkeit und abdominale Schmerzen während der Infusion sowie
- (Akro‑)Zyanose.

### Ausblick

Die Dokumentation von etwa 400 unselektierten Patienten mit CAD und CAS ist im Cold Agglutinin Disease Real World Evidence (CADENCE) Registry geplant. Dafür wurden im zweiten Quartal 2022 bereits die ersten Patienten rekrutiert [31]. Es handelt sich um eine internationale, prospektive Beobachtungsstudie in Europa und Nordamerika. In einer Substudie wird bei mindestens 30 Patienten auch die Wirksamkeit und Sicherheit einer Therapie mit Sutimlimab im klinischen Alltag dokumentiert. Die Rekrutierungsdauer wird etwa 3 Jahre betragen, und der letzte eingeschlossene Patient wird dann noch für weitere 3 Jahre beobachtet (geplante Gesamtdauer etwa 6 Jahre).

## Kasuistiken

Die folgenden Kasuistiken sollen beispielhaft den klinischen Verlauf, die Diagnostik und verschiedene therapeutische Überlegungen illustrieren. Sie sind für ein Expertentreffen aufbereitet und in diesem Rahmen diskutiert worden.

### Kasuistik 1 (Aschersleben)

- 76 Jahre alter, männlicher Patient in sehr schlechtem Allgemeinzustand. Diagnose der CAD bereits 2016. Monospezifischer DAT für C3d (++), Kälteagglutinintiter bei 4 °C 1:128, Knochenmarkbiopsie ohne Hinweis auf ein Non-Hodgkin-Lymphom. Alle B‑Zell-gerichteten Therapien hatten sich bis dato weitgehend ineffektiv im Hinblick auf die Aktivität der Hämolyse gezeigt.

Der Patient präsentierte sich bei Übernahme 2022 mit Luftnot, allgemeiner Schwäche, Reduktion der Muskelmasse und stark reduziertem Allgemeinzustand. Sein Eastern-Cooperative-Oncology-Group(ECOG)-Status wurde auf 3–4 geschätzt.

Schon mit Beginn der Sutimlimabtherapie (7500 mg bei Körpergewicht > 75 kg) ergab sich eine deutliche Besserung der Laborparameter innerhalb weniger Wochen mit einem deutlichen Abfall der Retikulozyten (von 221,00 auf 55,0 Gpt/l), einem schnellen Abfall des Bilirubins (von 62,10 auf 12,70 µmol/l), aber einem Anstieg des freien Hb (von 266 auf 1543 g/l). Die Laborwertveränderungen gingen mit einer deutlichen klinischen Verbesserung einher.

#### Kernergebnis.

Die Bestimmung des freien Hb ist möglicherweise anfällig und problematisch.

Die Notwendigkeit der Bestimmung des freien Hb wurde kontrovers diskutiert. Der Wert ist sehr anfällig bzw. empfindlich bei einer Halbwertszeit von etwa 1 h. Im direkten Vergleich sind die Bilirubin- und Haptoglobinwerte deutlich aussagekräftiger für das Ausmaß der Hämolyse und den Verlauf.

### Kasuistik 2 (Bonn)

- 81-jährige Patientin mit einer AIHA-Diagnose seit 2011, aufgrund derer seinerzeit eine Therapie mit Prednisolon erfolgte. Der erstmalige dokumentierte Nachweis von Kälteagglutininen gelang 2013. Eine Knochenmarkpunktion und bildgebende Untersuchungen ergaben keinen Nachweis eines Lymphoms. Hier wie auch später in den Jahren 2020–2022 erfolgten Therapiezyklen mit Rituximab, unter anderem auch in Kombination mit Bendamustin. Zusätzlich erfolgte 2022 eine Therapie mit Bortezomib.

Im Dezember 2022 stellte sich die Patientin dann erneute vor, mit positivem monospezifischem DAT für C3d und einem Kälteagglutinintiter von 1:128. Klinisch präsentierte sich die Patientin mit Fatigue, einer Raynaud-Symptomatik und einer Akrozyanose vor allem in den Wintermonaten. Unter der daraufhin begonnenen Sutimlimabtherapie kam es zu einem raschen Ansprechen der Hämolyseparameter (Abfall des Bilirubin- und Laktat-Dehydrogenase[LDH]-Werts, Anstieg des Hb-Werts) und einer deutlichen Verbesserung des Allgemeinzustands. Allerdings zeigte die Patientin im Verlauf der Therapie zunehmende Kopfschmerzen, deren individuelle Bedeutung das Ausmaß der Anämie überwog. Auf Wunsch der Patientin wurde Sutimlimab Ende Mai 2023 abgesetzt, seitdem verschlechtern sich die Werte wieder langsam.

#### Kernergebnis.

Die Kopfschmerzen könnten eine Folge der Komplementinhibition oder auch eine Nebenwirkung einer Blutdruckveränderung sein.

Während einer Therapie mit Sutimlimab auftretende unerwünschte Symptome können die Bereitschaft zu einer fortgesetzten Therapie einschränken. In der Diskussion wurde darauf hingewiesen, dass Kopfschmerzen typischerweise zu Beginn einer terminalen Komplementinhibition auftreten können, aber im Verlauf ansonsten nicht häufiger beobachtet werden. Zudem kann es unter Komplementinhibition und Verbesserung der Anämie zur Hypertonie mit konsekutivem Kopfschmerz kommen. Eine Kontrolle der Blutdruckwerte und Blutdruckeinstellung sowie die Überprüfung der Begleitmedikation wurden als Option empfohlen.

### Kasuistik 3 (Hamburg)

- 60-jährige Patientin mit Erstdiagnose einer CAD im Jahr 2018. Monospezifischer DAT für C3d positiv, Kälteagglutinintiter 1:2048, in Knochenmarkbiopsie 2 % B‑Zellen mit aberrantem Immunphänotyp und Leichtkettenrestriktion. Klinisch kam es zu Missempfindungen an den Fingerspitzen bei Kälteexposition, Abgeschlagenheit, Belastungsdyspnoe und dunklem Urin.

Die Therapie mit Sutimlimab wurde Mitte 2023 begonnen, einhergehend mit einer deutlichen Verbesserung der Laborwerte und des Allgemeinzustands. Allerdings war die Wirkung auf die Raynaud-Symptomatik unzureichend, sodass im Folgenden ergänzende Therapieoptionen diskutiert wurden.

#### Kernergebnis.

Bestehen trotz Therapie mit Sutimlimab klinische Beschwerden, können ergänzende Therapien erwogen werden.

Um der Akrozyanose und Raynaud-Symptomatik zu begegnen, wurde die zusätzliche Gabe von Rituximab allein oder in Kombination mit Bendamustin erwogen. Grundsätzlich wurde in der Entscheidung für eine ergänzende Therapie der individuelle Leidensdruck der Patientin in den Vordergrund gestellt.

### Kasuistik 4 (Bayreuth)

- 55-jährige Patientin mit bekanntem Raynaud-Syndrom seit 1986. Diagnose einer AIHA mit Kälteagglutininen im Jahr 2021. Klonale B‑Zell-Lymphozytose vom Nicht-B-CLL-Typ (*CLL* chronische lymphatische Leukämie). Verschiedene Therapieversuche mit Prednison und später Rituximab. Zuletzt mehrfache Hämolyseschübe. In der Diagnostik 2022 dann Hämolyse, Kälteantikörper mit Spezifität für I-Anitgen, positiver monospezifischer DAT für C3d, Kälteagglutinintiter bei 4 °C 1:512, kein Anhalt für eine maligne Erkrankung. Klinisch ausgeprägte Fatigue (Patientin konnte maximal 5–6 Treppenstufen ohne Pause zurücklegen) und Muskelschmerzen

Mit Beginn der Sutimlimabtherapie normalisierten sich die Laborwerte und die Arbeitsfähigkeit konnte wiederhergestellt werden. Es bestand eine residuale Raynaud-Symptomatik bei Kälte. Die Patientin klagte jedoch über „zu viel Energie“. Berufsbedingt wurden die Therapieintervalle auf 3 Wochen verlängert, später erfolgte eine Therapiepause von 6 Wochen mit Anstieg der Hämolyseparameter. Nach Neubeginn der Sutimlimabtherapie normalisierten sich die Laborwerte wieder.

#### Kernergebnis.

Eine Verlängerung der Therapieintervalle kann in Ausnahmefällen mit entsprechenden Kontrollen erwogen werden.

Am Beispiel dieser Patientin wurde die Bedeutung der Therapieintervalle erörtert. Während medizinisch die Einhaltung der 2‑wöchigen Intervalle geboten scheint (erneute Aufsättigung ab Tag 17), entwickeln manche Patienten eine gewisse Therapiemüdigkeit und sind schwer zu einem entsprechenden Rhythmus zu bewegen. Eine großzügige Antibiose bei Hinweisen auf eine bakterielle Infektion als möglicher zusätzlicher Auslöser einer hämolytischen Krise für solche Fälle wurde empfohlen. Man müsse in jedem Fall den Patienten entsprechend aufklären und im Hinblick auf eine mögliche Durchbruchhämolyse sensibilisieren. Es wurde davon ausgegangen, dass bei Durchbruchsymptomatik die Wiederaufnahme der Therapie mit Sutimlimab erfolgversprechend ist.

### Kasuistik 5 (Ulm)

- 81-jährige Patientin mit einer CAD-Diagnose aus dem Jahr 2010, allerdings ohne Daten zu DAT, Kälteagglutinintiter und Knochenmarkbiopsie. In der Vorgeschichte Thromboembolien (2012), Adenokarzinom des Rektums und des Endometriums (2021), Vorhofflimmern seit 2021 und Diagnose einer hochgradigen Trikuspidal- (2021) und Mitralinsuffizienz (2022). Zunächst Therapie mit Eculizumab im Rahmen der DECADE-Studie (2012), später mit Rituximab (2013) und dann (partiell überlappend) stabile Therapie mit Cyclophosphamid über 4 Jahre (2013–2017). Erneute Therapie mit Rituximab. Anfang 2023 Verdacht auf Rezidiv des Rektumkarzinoms und Verdacht auf gastrointestinale Blutung Mitte des Jahres

Mitte 2023 wurde die Therapie mit Sutimlimab eingeleitet, mit insuffizientem Anstieg des Hb-Werts und weiterhin erhöhten Bilirubin- und LDH-Werten. Bei einem Ferritinspiegel von 34 µg/l war eine Eisensubstitution empfohlen. Hierunter zeigte sich im Verlauf eine Besserung der Hb-Werte.

#### Kernergebnis.

Komplexe Komorbidität, Diagnostik und Diagnosekriterien sollten bei Verdacht auf eine eingeschränkte Wirksamkeit überprüft werden.

**Abb. 4:**
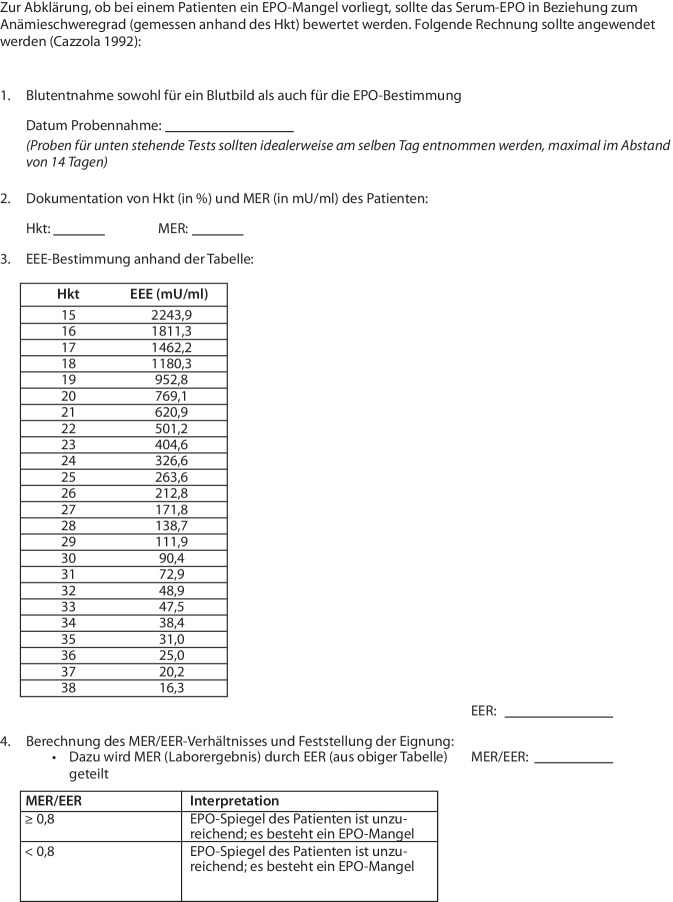
Erfassung eines Erythropoetinmangels. *EER* „expected EPO result“ (erwarteter EPO-Spiegel), *EPO* Erythropoetin, *Hkt* Hämatokrit, *MER* „measured serum EPO“ (gemessener EPO-Spiegel im Serum). (Modifiziert nach [32])

Sinnvoll wäre zunächst die Ergänzung der Diagnostik mit monospezifischem DAT, Bestimmung des Kälteagglutinintiters und Knochenmarkdiagnostik, da entsprechende Daten aktuell nicht mehr vorliegen. Anschließend sollte der Ausschluss einer mechanisch induzierten Hämolyse erfolgen (→ Trikuspidal- und Mitralklappe mit Restinsuffizienz), dann eine Erythropoetindiagnostik (Abb. 4 im Anhang).

### Kasuistik 6 (Regensburg)

- 78-jähriger Patient, seit 2012 mit Akrozyanose bei Kälte und intermittierend mit dunklem Urin. Positiver DAT, Nachweis von Kälteautoantikörpern (1:32 bei 4 °C; zur Diagnose notwendiger Titer: 1:64). Keine pathologischen Lymphknoten. Phasenweise gut kompensierte Hämolyse mit Hb-Werten zwischen 8,5 und 13,2 g/dl. Karotisstenose und koronare Herzkrankheit als Begleiterkrankungen

Im Jahr 2022 erfolgte die erste Gabe von Rituximab in 4‑wöchentlichen Intervallen. Im Jahr 2023 dann wurde die Sutimlimabtherapie begonnen, mit protrahiertem Ansprechen des Hb unter den ersten 4 Gaben. Nach Eisensubstitution i.v. kam es zu einem deutlichen Hb-Anstieg bis auf 12,6 g/dl. Die Sutimlimabtherapie wurde nach 10 Gaben auf Wunsch des Patienten abgebrochen, der den Hb-Anstieg auf die Eisengabe zurückführte. In der Therapiepause fiel das Hb trotz fortgeführter Eisensubstitution erneut auf 10,5 g/dl ab (Retikulozyten 70 ‰), zudem entwickelten sich kälteagglutininassoziierte Symptome (Akrozyanose). Im Februar 2024 erfolgte die Wiederaufnahme der Sutimlimabtherapie mit raschem Verschwinden aller kälteagglutininassoziierten Symptome und Hb-Anstieg auf 13,0 mg/dl (Retikulozyten 30 ‰).

Letztendlich wurde die Diagnose revidiert. Es handelte sich um eine klassische CAD im Sinne einer lymphoproliferativen Erkrankung mit (kurzzeitigem) Nachweis eines kleinen B‑Zell-Klons, MYD88 negativ. Der Klon wurde initial als B‑CLL (bzw. monoklonale B‑Zell-Lymphozytose) fehlgedeutet.

#### Kernergebnis.

Mangelzustände (Ferritin, Vitamin B12 und Folsäure) sollten bei einer hämolytischen Erkrankung immer ausgeschlossen bzw. frühzeitig ausgeglichen werden.

## Resümee

Die CAD ist eine sehr seltene eigenständige Autoimmunerkrankung mit zugrunde liegender lymphoproliferativer B‑Zell-Erkrankung. Es kommt zur Aktivierung des klassischen Aktivierungswegs des Komplementsystems und konsekutiv zur extravasalen und zum Teil intravasalen Hämolyse. Eine Verschlüsselung der CAD ist unter dem ICD-10-Code D59.10 möglich.

Die Diagnosestellung erfordert den Nachweis einer chronischen Hämolyse, einen deutlichen Nachweis von C3d im DAT bzw. Coombs-Test, den Nachweis von Kälteagglutininen mit einem Titer ≥ 1:64 bei 4 °C und den Ausschluss einer malignen Erkrankung oder relevanten Infektion.

Die CAD ist mit Thromboembolien und möglicherweise mit einer erhöhten Mortalität assoziiert.

Therapeutisch steht neben dem Schutz vor Kälteeinwirkung, der ausreichenden Versorgung mit Folsäure, Vitamin B12 und Eisen bei entsprechendem Mangel, einer ausreichenden Hydratation bei Krisen und einer Thromboseprophylaxe in Risikosituationen insbesondere eine Komplementinhibition mit Sutimlimab im Vordergrund. Zusätzlich oder alternativ kann in bestimmten Situationen eine B‑Zell-depletierende Therapie mit Rituximab und Bendamustin erwogen werden.

Regelhaft findet sich ein schnelles Ansprechen auf Sutimlimab, wobei simultan Schutzimpfungen und gegebenenfalls eine Antibiotikaprophylaxe für 2 Wochen durchgeführt werden. Nach der Aufsättigung folgt die Erhaltungstherapie alle 2 Wochen. Der klinische Kontext (beispielsweise Eisenmangel, relativer Mangel an Erythropoetin) und das Vermeiden einer Kälteexposition müssen während der Therapie zur Erzielung eines optimalen Ergebnisses berücksichtigt werden. Insgesamt ist die Therapie mit Sutimlimab sicher und gut verträglich. Schwere Nebenwirkungen sind eher selten.

Die Wirksamkeit beim CAS ist bislang nicht systematisch untersucht, aber wahrscheinlich. Eine Zulassung von Sutimlimab für die Therapie des CAS liegt bislang nicht vor.

## Fazit für die Praxis

- Bei der Kälteagglutininerkrankung („cold agglutinin disease“ [CAD]) kommt es zur Aktivierung des Komplementsystems über den klassischen Aktivierungsweg und konsekutiv zur extravasalen und zum Teil intravasalen Hämolyse.
- Die Diagnosestellung erfordert den Nachweis einer chronischen Hämolyse, einen deutlichen Nachweis von C3d im monospezifischen direkten Antiglobulintest, den Nachweis von Kälteagglutininen mit einem Titer ≥ 1:64 bei 4 °C und den Ausschluss einer malignen Erkrankung oder relevanten Infektion.
- Die CAD ist mit Thromboembolien und möglicherweise mit einer erhöhten Mortalität assoziiert.
- Therapeutisch stehen im Vordergrund: Schutz vor Kälteeinwirkung, ausreichende Versorgung mit Folsäure, Vitamin B12 und Eisen bei entsprechendem Mangel, ausreichende Hydratation bei Krisen, Thromboseprophylaxe in Risikosituationen, Komplementinhibition mit Sutimlimab.
- Insgesamt ist die Therapie mit Sutimlimab sicher und gut verträglich. Schwere Nebenwirkungen sind eher selten.
